# Integrated use of polyphosphate and P-solubilizing bacteria enhanced P use efficiency and growth performance of durum wheat

**DOI:** 10.3389/fmicb.2023.1211397

**Published:** 2023-07-05

**Authors:** Said Khourchi, Wissal Elhaissoufi, Ammar Ibnyasser, Meryem Haddine, Rachid Ghani, Youssef Zeroual, Pierre Delaplace, Adnane Bargaz

**Affiliations:** ^1^Agrobiosciences Program, College for Sustainable Agriculture and Environmental Sciences, , Mohammed VI Polytechnic University (UM6P), Ben Guerir, Morocco; ^2^TERRA – Teaching and Research Center, Plant Sciences, Gembloux Agro-Bio Tech, Université de Liège, Gembloux, Belgium; ^3^Situation Innovation, OCP Group, Jorf Lasfar, El Jadida, Morocco

**Keywords:** phosphate solubilizing bacteria, polyphosphates, phosphatases, rhizosphere, P use efficiency, chlorophyll fluorescence

## Abstract

Coupling phosphate-solubilizing bacteria (PSB) with P fertilizers, including polyphosphates (PolyP), was reported as eco-efficient approach to enhance P use efficiency. Although PSB have been recently reported to hydrolyze PolyP, the plant growth promoting mechanisms of PolyP-PSB co-application were not yet uncovered. This study aims to evaluate the effect of a PSB consortium (PSB_Cs_) on growth, P use efficiency (PUE), and wheat yield parameters under PolyP (PolyB) application. Co-application of PolyB-PSB_Cs_ significantly enhanced wheat growth at 75 days after sowing (DAS) compared to 30 DAS. A significant increase in shoot dry biomass (47%), shoot inorganic P content (222%), PUE (91%), and root P absorption efficiency (RPAE, 99%) was noted compared to unfertilized plants. Similarly, the PolyB-PSB_Cs_ co-application enhanced morphological root traits at 30 DAS, while acid phosphatase activities (root and rhizosphere), RPAE, and PUE were significantly increased at 75 DAS. The improved wheat P acquisition could be attributed to a lower investment in root biomass production, and significant induction of acid phosphatase activity in roots and rhizosphere soil under PolyB-PSB_Cs_ co-application. Consequently, the PolyB-PSB_Cs_ co-application significantly improved aboveground performance, which is reflected by increased shoot nutrient contents (P 300%, K 65%), dry weight (54%), and number (50%) of spikes. Altogether, this study provides relevant evidence that co-application of PolyP-PSB_Cs_ can be an integrated and environmentally preferred P fertilization approach owing to the dual effects of PolyP and PSB_Cs_ on wheat PUE.

## 1. Introduction

Sustaining agricultural food production is heavily dependent on suitable phosphorus (P) supply, among other nutrient, due to its direct and essential role in crop growth and yield (Lynch, 2007; Lambers et al., 2011; Shen et al., 2011). However, the low mobility and high adsorption of P (especially orthophosphate (OrthoP) ions) onto soil mineral surfaces results in low P fertilizer use efficiency (PFUE) and consequently limited crop productivity (Richardson and Simpson, 2011; Rutkowska et al., 2014; Secco et al., 2017). The limited PFUE called for the development of more efficient P fertilizers that are less adsorbed/easily precipitated with soil minerals and can induce positive root/rhizosphere responses, allowing better P use efficiency by the plant (Marcelino et al., 2016; Naz and Sulaiman, 2016; Wang et al., 2016; Hamilton et al., 2018; Honvault et al., 2021).

In that regard, polyphosphates (PolyP) were developed as P polymeric fertilizers characterized by their lower susceptibility to adsorption (onto mineral oxides and clays), and likely progressive hydrolysis during plant growth, as well as their potential effects on root growth (McBeath et al., 2005; Torres-Dorante et al., 2006; Gao et al., 2020; Kusi et al., 2021; Chtouki et al., 2022; Khourchi et al., 2022b). The agronomic use efficiency of PolyP was highly dependent on their rate of hydrolysis, which was governed by intricate interactions of soil and plant related factors (i.e., physicochemical: pH, temperature, soil moisture, etc., and biological: microbial and root activities) (Dick and Tabatabai, 1987; McBeath et al., 2007; Chtouki et al., 2022; Khourchi et al., 2022a, 2023).

Considering the foraging potential of roots and associated microbes in mobilizing and acquiring P, the roots and microbial activities in the rhizosphere interface are of significant importance to enhance PolyP use efficiency. In this context, P-solubilizing bacteria (PSB) were reported to significantly hydrolyze PolyP with different chain length through producing P-hydrolyzing enzymes (acid phosphatases (APase) and pyrophosphatases) and acidifying the rhizosphere (pH lowering and production of organic acids) (Dick and Tabatabai, 1987; Khourchi et al., 2022a). Furthermore, a PSB consortium (PSB_Cs_) showed the highest PolyP hydrolysis rate compared to individual PSB species, which is likely explained by significant amounts of organic acids, APase, and pyrophosphatases exuded by PSB_Cs_ under PolyP application (Khourchi et al., 2022a). However, the research gap regarding the potential contribution of PSB as well as the microbial mechanisms involved in PolyP use efficiency in plant–soil continuum remain unaddressed (Khourchi et al., 2023), with only one published study by Khourchi et al. (2022a). Hence, the present study is among the first to evaluate the effects of PSB-PolyP co-application on PUE and plant growth performance.

In this study, we (i) investigate the crucial role of PSB in enhancing the P acquisition and PUE of wheat plants under PolyB application, (ii) study the role of PolyB-PSB_Cs_ co-application in modulation of trade-offs between the morphological root traits, root biomass, and P-hydrolyzing enzyme activities (roots and rhizosphere soil) for better P acquisition, and (iii) evaluate the effects of PolyB-PSB_Cs_ co-application on above-and below-ground parameters interactions allowing a better wheat morpho-physiological performance.

## 2. Materials and methods

The present study was performed to assess the plant growth effects of PolyB-PSB_Cs_ co-application on wheat [*Triticum turgidum* subsp. Durum, variety ‘Karim’] growth performance at two growth stages (Z22 and Z57 corresponding to 30-and 75-days after sowing (DAS), respectively) under PolyB application. The four PSB forming the consortium were identified as *Bacillus siamensis, Rahnella aceris, Pantoea hericii,* and *Bacillus paramycoides* (Khourchi et al., 2022a). The PSB_Cs_ used showed a high capacity to hydrolyze PolyP with different chain lengths (including PolyB: a short-chain PolyP) through producing P-hydrolyzing enzymes (APase and pyrophosphatases) and organic acids, as the main bacterial mechanisms involved in PolyP hydrolysis. In addition, the four PSB exhibited other beneficial plant growth promoting traits (e.g., tri-calcium P and rock P solubilization, nitrogen fixation, auxin production, etc.) (Khourchi et al., 2022a).

The inoculum of PSB_Cs_ was prepared as described by Khourchi et al. (2022a). Briefly, each PSB species was cultivated individually in tryptone soya broth (TSB) and incubated on a rotary shaker (150 rpm) at 28°C for 24 h. The bacterial pellet was collected by centrifugation and washed with sterilized distilled water. The PSB_Cs_ inoculum was prepared by mixing equal proportions of the four PSB species. Then, the cell density was adjusted to 10^8^ CFU/ml. The inoculation was performed by soaking disinfected seeds in the inoculum for 2 h under continuous shaking (90 rpm) at room temperature.

### 2.1. Experimental design and plant growth conditions

In this study, two sets of experiments (30 and 75 DAS) were conducted under greenhouse conditions (similar conditions described by Khourchi et al., 2022b) at the UM6P experimental farm, according to a completely randomized design. A P-deficient substrate (6 ppm of available P) composed of a sterile mixture of soil, sand, and peat (2:0.5:0.5, v:v:v, respectively), was used for the two experiments.

Wheat seeds were surface disinfected by soaking in sodium hypochlorite (6% for 5 min) and ethanol (96% for 1 min) followed by five washes with sterile distilled water. The disinfected seeds were then inoculated as described above, while the uninoculated seeds were soaked in sterile distilled water for 2 h.

The inoculated and uninoculated wheat seeds were grown in above mentioned substrate for both 30 DAS and 75 DAS. The P was applied as PolyB (a short-chain PolyP fertilizer) and OrthoP fertilizer at the rate of 60 kg P/ha. The N, K, and micro-nutrients were balanced for all treatments and supplied as described by Khourchi et al. (2022b). The experiments consisted of four treatments, with eight replicates per treatment, including plants inoculated with PSB_Cs_ and fertilized with PolyB, uninoculated plants fertilized with PolyB, uninoculated plants fertilized with OrthoP (readily available P), and uninoculated and unfertilized plants (no P application) abbreviated as PolyB-PSB_Cs_, PolyB, OrthoP, and P0, respectively. The plants were watered twice a week with distilled water to keep the soil moisture at approximately 60% of water-holding capacity.

### 2.2. Measurements of above- and below-ground traits

At harvest, the roots were carefully cleaned to remove soil particles and separated from shoots. The rhizosphere soils were harvested by collecting the soils that were tightly adhered to the roots. Fresh samples of shoots, roots, and soil were stored at −20°C for biochemical analyses (e.g., inorganic P (Pi) content, APase activity, available P content, and soil microbial biomass P). The plants were oven dried at 80°C for 72 h for dry weight measurements of shoots (SDW) and roots (RDW).

#### 2.2.1. Shoot and root inorganic P contents

Samples of shoots and roots were ground in cold sodium acetate buffer (0.2 M, pH 5.6). Plant extract was centrifuged at 12000 × *g* for 10 min at 4°C and the resultant supernatant (50 μL) was used for quantification of Pi and determination of APase activity. The shoot and root Pi contents were determined spectrophotometrically using the molybdate blue method (Khourchi et al., 2022b).

#### 2.2.2. Morphological root traits

The washed roots were used to measure the morphological root traits (root length (RL), root surface area (RSA), root volume (RV), and root diameter (RD)). Roots were gently spread on a flat tray filled with a shallow layer of water and then placed into an Epson flatbed scanner with a resolution of 300 dpi. The above morphological root traits in response to the PolyB-PSB_Cs_ co-application were auto-quantified using WinRhizo software (Regent Instruments Inc., Québec, QC, Canada).

#### 2.2.3. Soil available P concentration and soil microbial biomass P

The rhizosphere soil available P was measured as described by Khourchi et al. (2022b). An aliquot of soil (0.5 g) was placed into 10 ml of sodium bicarbonate (0.5 M, pH 8.5). The mixture was stirred for 30 min at 150 rpm and filtered. The filtrate was used to determine the available P following the spectrophotometric molybdate blue method as described above.

Microbial biomass P (MBP) was determined using the chloroform fumigation-extraction method (Corstanje et al., 2007). Briefly, two samples (2 g of fresh soil) of moist soil were fumigated with chloroform (ethanol-free) under 200 mbar vacuum pressure for 18 h. In parallel, non-fumigated soil samples were processed under similar conditions (200 mbar for 18 h), without chloroform. The MBP concentrations were determined as the difference between the NaHCO-extractable P (determined as described above) concentrations in the fumigated and non-fumigated soils, divided by the extraction efficiency factor (K_EP_ = 0.40) as follows:


$$\mathrm{MBP}\;\left(\mathrm{ppm}\right)=\Delta P/K_{\mathrm{EP}}$$

MBP (ppm): microbial biomass P, ΔP: (NaHCO-extractable P in fumigated soil) – (NaHCO-extractable P in non-fumigated soil), and K_EP_: The extraction efficiency factor.

#### 2.2.4. Phosphorus use efficiency parameters

Several PUE related parameters were calculated based on plant P accumulation, plant biomass, root traits, and rhizosphere soil available P to estimate the contribution of PSB_Cs_ inoculation to enhanced availability and acquisition of P from PolyP. The P use efficiency was defined as the ratio of shoot dry biomass to shoot P content (Cai et al., 2020). The root P acquisition efficiency (RPAE) was calculated as the amount of P taken up per unit of root dry weight (Khourchi et al., 2022a). This calculated parameter indicates the capacity of the wheat root to absorb P from the soil. The PFUE was calculated using the following equation (Gao et al., 2020):


$$\mathrm{PFUE}=\left(P\;{\mathrm{content}}_{P-\mathrm{fertilized treatment}}-P\;{\mathrm{content}}_{\mathrm{unfertilized treatment}}\right)/\mathrm{applied}\;P$$

As RL is one of the morphological root traits that is most responsive to P availability and P acquisition, shoot Pi content to RL and nutrient (total shoot N, P, and K) contents to RL ratios were calculated to assess the effects of PolyB-PSB_Cs_ co-application on root traits involved in PUE and nutrient acquisition.

#### 2.2.5. Acid phosphatase activities in rhizosphere soil and root tissues

The rhizosphere soil and root APase activities were measured using the *p*-nitrophenyl phosphate (*p*-NPP)-based method (Khourchi et al., 2022b). An aliquot of fresh soil or fresh roots were supplied to cold acetate buffer and *p*-NPP as APase substrate. The reaction was stopped, after 1 h of incubation at 37°C, by adding CaCl_2_ and NaOH. The APase activity (nmol *p*-NPP/g/h) was determined by quantifying the intensity of the yellow color using a UV–Vis spectrophotometer at 405 nm.

#### 2.2.6. Shoot nutrients (N, P, and K) contents and yield related parameters

At 75 DAS, shoot nutrient (N, P, and K) contents and wheat yield parameters (spike dry weight, spike number, spike N, P, and K contents) were determined. The total N, P, and K contents were measured in shoots and spikes using dried and finely ground shoots and spikes. The shoot and spike powders were digested using nitric acid and analyzed for P and K contents using inductively coupled plasma optical emission spectrometry (Agilent 5,110 ICP-OES, USA). The total N content (shoots and spikes) was determined following the Kjeldahl method (KjelMaster K-375, Netherlands) (Khourchi et al., 2022b).

#### 2.2.7. Photosynthesis related parameters

During 75 days of wheat growth (Z57), both the chlorophyll content index (CCI) and chlorophyll ‘a’ fluorescence were measured *in situ*. The CCI was measured non-destructively using a portable chlorophyll-meter (Chlorophyll Content Meter, model CL-01, Hansatech Instruments). Measurement of chlorophyll ‘a’ fluorescence was performed using a portable fluorometer (Plant Efficiency Analyzer, Hansatech Instruments Ltd). Prior to each measurement, the leaves were adapted to dark for 15 min using black leaf clips as described by Dewez et al. (2018). The maximum quantum yield (Fv/Fm) and the performance index (PI) were used in this study as the most relevant parameters to assess the potential efficiency of the photosynthetic apparatus.

### 2.3. Statistical analysis

The statistical data analyses were performed using Minitab software (version 21.1.0). Two-way analyses of variance were used to evaluate the effects of PolyP and PSB_Cs_ on the wheat above-and below-ground parameters. Tukey *post hoc* test was used to determine the significant difference between the means of the treatments at 0.05 significance level.

## 3. Results

### 3.1. Effect of PolyP-PSB consortium co-application on plant biomass and Pi content

**Figure 1:**
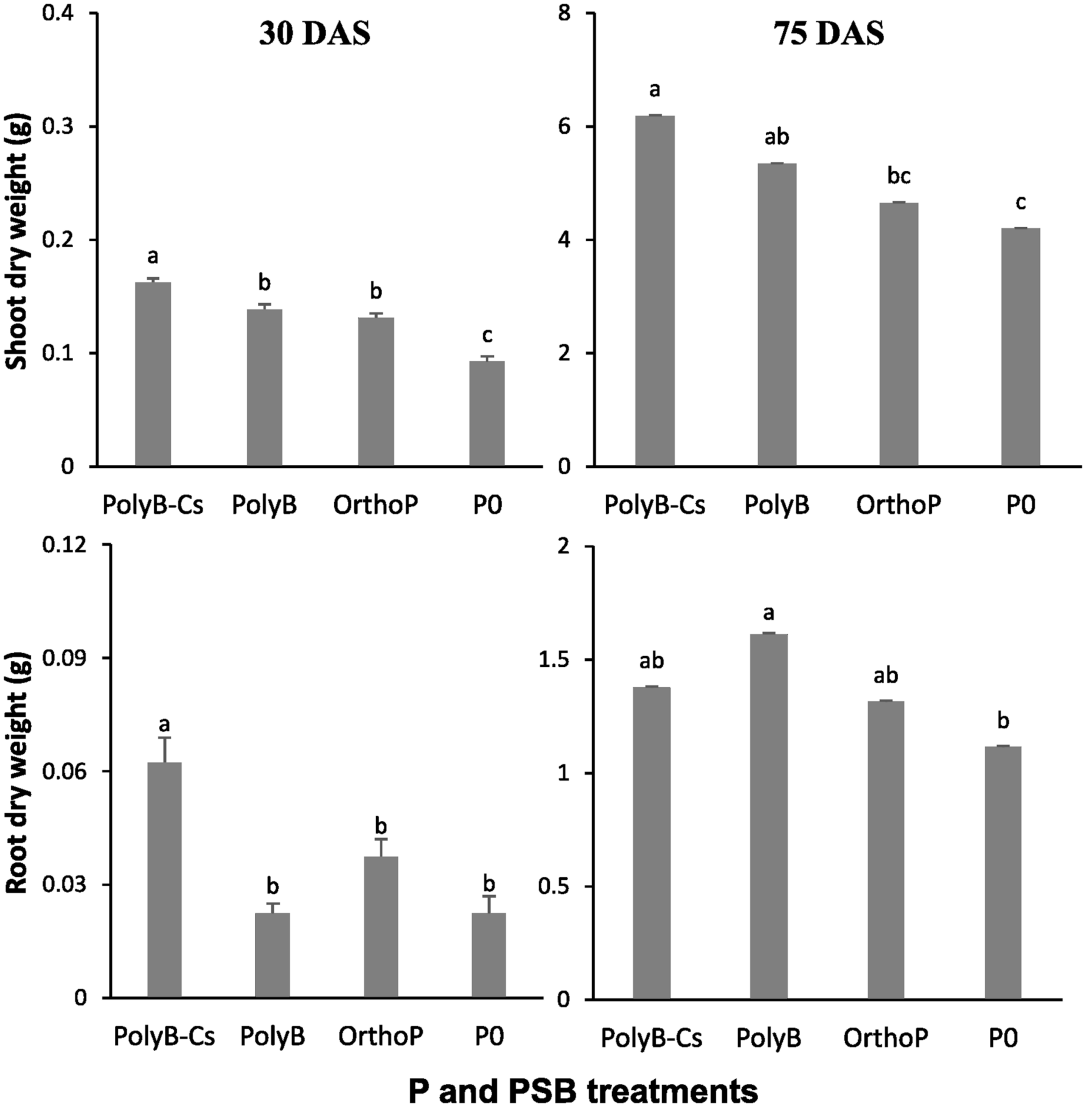
Effects of PSB consortium and PolyB co-application on root and shoot dry weights of wheat plants at 30 and 75 days after sowing. Data are mean values ± SD (*n* = 8). Different lowercase letters above the bars indicate significant differences (*p* < 0.05) according to Tukey’s test. PolyB-Cs, Polyphosphate application combined with a consortium of four P-solubilizing bacteria.

The PolyP-PSB_Cs_ co-application significantly enhanced shoot and root biomass compared to unfertilized plants (Figure 1). The PolyB-PSB_Cs_ co-application significantly enhanced the SDW (75 and 47%) and RDW (178 and 25%) at both 30 and 75 DAS, respectively compared to unfertilized plants, while the unfertilized plants showed the lowest SDW and RDW. The SDW and RDW under PolyP-PSB_Cs_ co-application were significantly higher at 75 DAS compared to 30 DAS. Interestingly, the shoot biomass under PolyB (alone or in combination with PSB) was significantly increased at both 30 and 75 DAS compared to OrthoP-fertilized and unfertilized plants.

**Figure 2:**
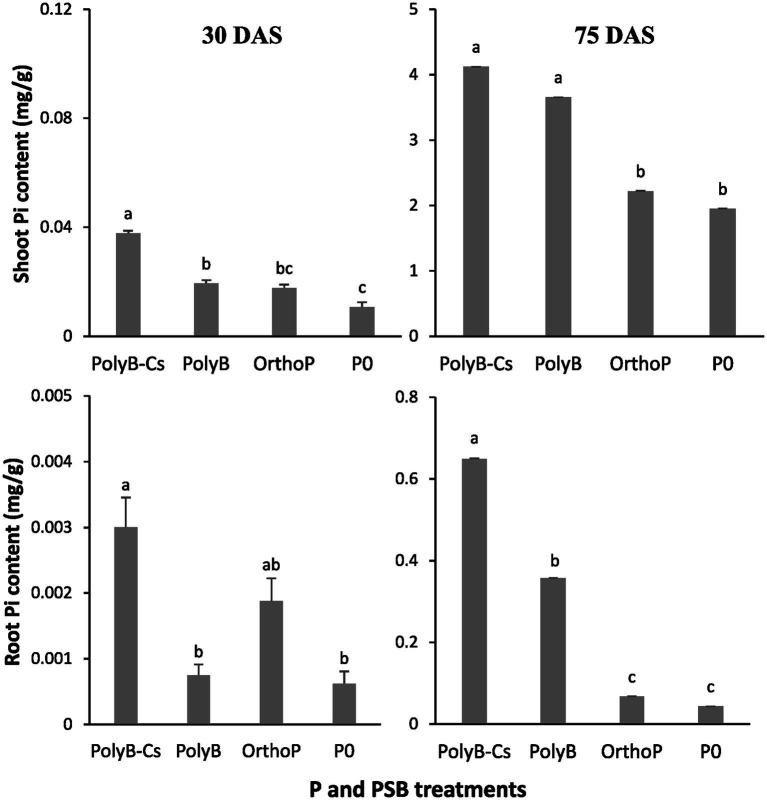
Effects of PSB consortium and PolyB co-application on shoot and root Pi contents of wheat plants at 30 and 75 days after sowing. Data are mean values ± SD (*n* = 8). Different lowercase letters above the bars indicate significant differences (*p* < 0.05) according to Tukey’s test. PolyB-Cs, polyphosphate application combined with a consortium of four P-solubilizing bacteria.

The PolyB-PSB_Cs_ co-application significantly increased shoot and root Pi contents at 30 and 75 DAS (Figure 2). The shoot Pi contents significantly increased by 244 and 112% in response to co-application of PolyB-PSB_Cs_ at 30 and 75 DAS, respectively, compared to unfertilized and uninoculated plants. Similarly, the root Pi content was 5- (30 DAS) and 15-times (75 DAS) higher in response to PolyB-PSB_Cs_ co-application than unfertilized and uninoculated plants. Similar to dry biomass, the improved Pi contents was more pronounced at 75 DAS, where both shoot and root Pi contents were 214-and 110-times higher from 30 to 75 DAS in response to PolyB-PSB_Cs_ co-application, compared to uninoculated and unfertilized plants. Moreover, except shoot Pi content at 75 DAS, the PolyB-PSB_Cs_ co-application significantly enhanced root and shoot Pi contents at both stages compared to PolyB alone.

### 3.2. Effect of PolyP-PSB consortium co-application on root morphological traits

Our findings indicate that morphological root traits were significantly influenced in response to PolyB-PSB_Cs_ co-application depending on growth stages (Table 1). At 30 DAS, PSB_Cs_ significantly increased the RL, RSA, and RV, along with narrower RD compared to uninoculated treatments (Table 1). For instance, PolyB-PSB_Cs_ co-application significantly increased RL (137%), RSA (131%), and RV (125%) compared to uninoculated and unfertilized plants. At 75 DAS, however, the unfertilized control plants showed the highest values for morphological traits such as RL, RSA, and RV, and lowest RD compared to plants under PolyB-PSB_Cs_ co-application, especially RL (Table 1). The uninoculated and unfertilized plants showed the highest RL (52%) and RSA (19%) compared to inoculated plants. The RD at 75 DAS was significantly increased in response to PolyB-PSB_Cs_ co-application and OrthoP application compared to uninoculated and unfertilized plants.

**Table 1:** Effects of PSB consortium and PolyB application on morphological root traits (RL, root length; RSA, root surface area; RD, root diameter; and RV, root volume) of wheat plants at 30 and 75 days after sowing.

	RL (m)		RSA (cm^2^)		RD (mm)		RV (cm^3^)	
	30 DAS	75 DAS	30 DAS	75 DAS	30 DAS	75 DAS	30 DAS	75 DAS
PolyB-Cs	0.66^a^	2.81^c^	63.98^a^	357.01^a^	0.31^b^	1.21^a^	0.5^a^	3.68^a^
PolyB	0.25^b^	3.89^ab^	28.78^b^	420.88^a^	0.38^a^	1.06^b^	0.27^b^	3.68^a^
OrthoP	0.27^b^	3.23^bc^	30.5^b^	359.99^a^	0.36^a^	1.09^ab^	0.28^b^	3.21^a^
P0	0.28^b^	4.28^a^	27.66^b^	424.76^a^	0.32^b^	0.96^b^	0.22^b^	3.4^a^

PolyB-Cs, polyphosphate application combined with a consortium of four P-solubilizing bacteria. Data are means (*n* = 8). Different letters indicate a significant difference according to Tukey’s test: *p* < 0.05.

### 3.3. Effect of PolyP-PSB consortium co-application on P use efficiency

**Figure 3:**
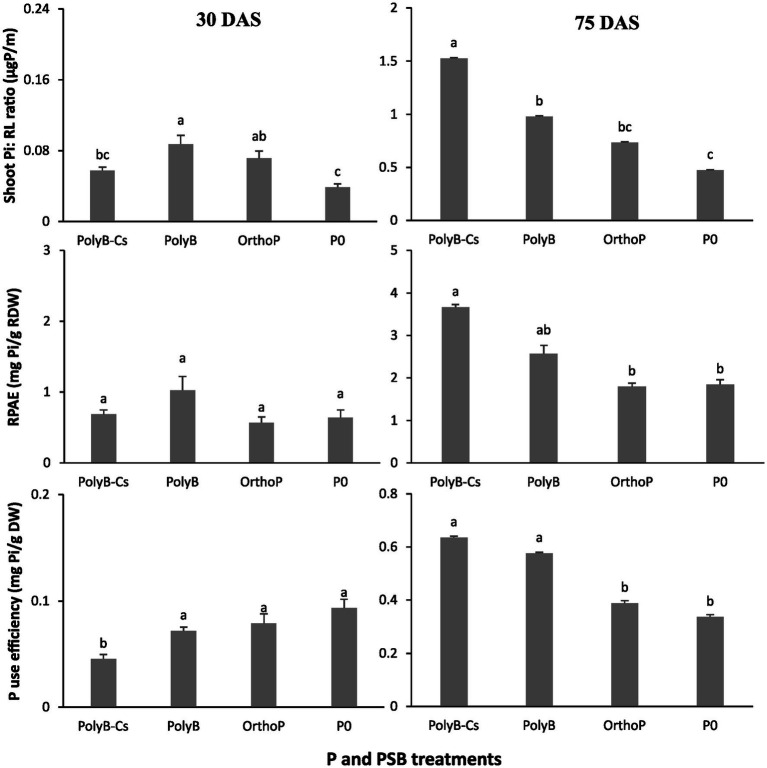
Effects of PSB consortium and PolyB co-application on shoot Pi to root length ratio, root P acquisition efficiency and P use efficiency of wheat plants at 30 and 75 days after sowing. Data are mean values ± SD (*n* = 8). Different lowercase letters above the bars indicate significant differences (*p* < 0.05) according to Tukey’s test. PolyB-Cs, polyphosphate application combined with a consortium of four P-solubilizing bacteria.

**Figure 4:**
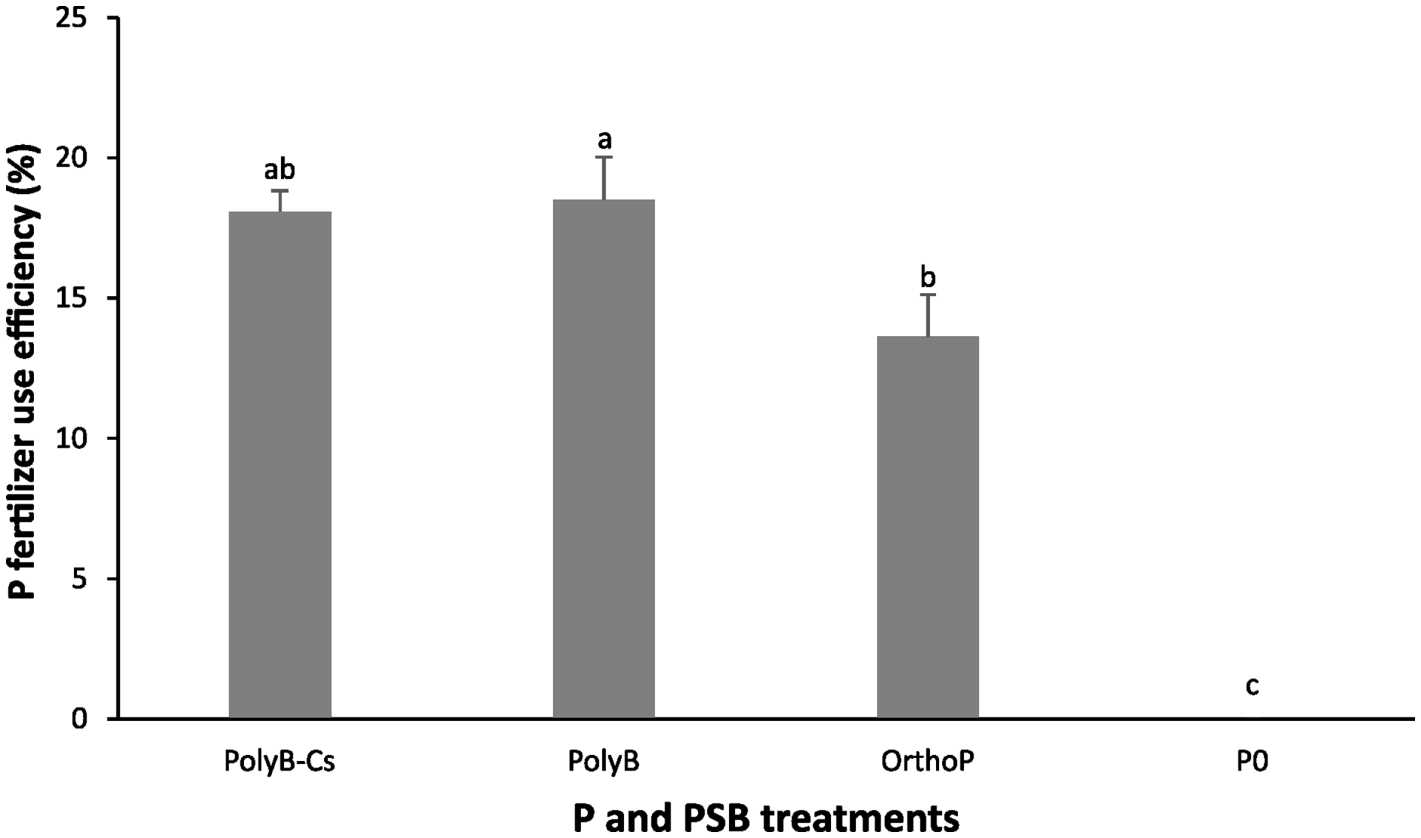
Effects of PSB consortium and PolyB co-application on P fertilizer use efficiency of wheat plants at 75 days after sowing. Data are mean values ± SD (*n* = 8), Different lowercase letters above the bars indicate significant differences (*p* < 0.05) according to Tukey’s test. PolyB-Cs, polyphosphate application combined with a consortium of four P-solubilizing bacteria.

Our findings showed that PolyB-PSBCs co-application significantly increased wheat P acquisition by enhancing several PUE related parameters such as shoot Pi: RL ratio, RPAE, PUE, and PFUE (Figures 3, 4). The co-application of PolyB-PSB_Cs_ significantly improved shoot Pi: RL ratio, P use efficiency, and root P absorption efficiency (Figure 3). This improvement was more pronounced at 75 DAS than 30 DAS. Indeed, no significant difference was observed in PUE parameters between the four treatments at 30 DAS, while the inoculated plants at 75 DAS showed remarkable improvement in shoot Pi: RL ratio, RPAE, and PUE compared to uninoculated and OrthoP-fertilized plants. For instance, the co-application of PolyB-PSB_Cs_ increased the shoot Pi: RL ratio, RPAE, and PUE by 222, 99, and 88%, respectively, compared to unfertilized plants. Despite the improvement in PUE and RPAE in response to PSB inoculation, the difference remains non-significant compared with PolyB alone. In addition, the PFUE was significantly improved in co-application of PolyB-PSB_Cs_ and PolyB alone compared to OrthoP application (Figure 4). Hence, PFUE increased by 32 and 36% in response to PolyB-PSB_Cs_ co-application and PolyB alone compared to OrthoP application.

### 3.4. Effect of PolyP-PSB consortium co-application on soil P availability, and acid phosphatases in root and rhizosphere soil

**Figure 5:**
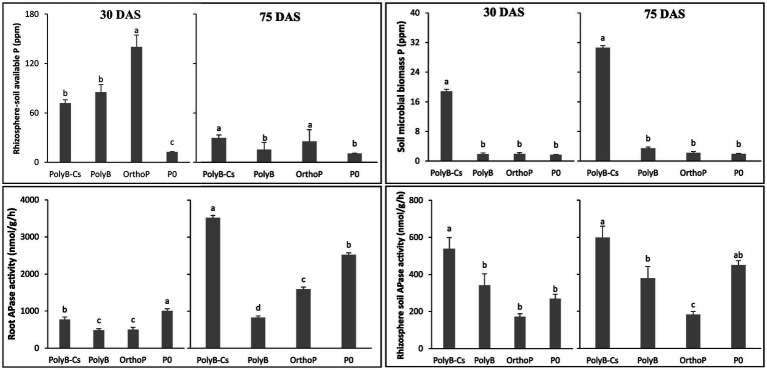
Effects of PSB consortium and PolyB co-application on rhizosphere soil available P, microbial biomass P, acid phosphatases activity in the rhizosphere soil and the roots of wheat plants at 30 and 75 days after sowing. Data are mean values ± SD (*n* = 8). Different lowercase letters above the bars indicate significant differences (*p* < 0.05) according to Tukey’s test. PolyB-Cs, polyphosphate application combined with a consortium of four P-solubilizing bacteria.

Given the involvement of PSB in increasing P availability from PolyP, our results showed that available P in rhizosphere soil under PolyB-PSB_Cs_ co-application was significantly increased and statistically similar to that of OrthoP (readily available P) at 75 DAS, while OrthoP application showed the highest available P content in the rhizosphere soil compared to PolyB application at 30 DAS. Moreover, the soil MBP was remarkably increased in the rhizosphere of inoculated plants at 30 and 75 DAS (Figure 5).

At 30 DAS, the available P was two-times higher in response to OrthoP application compared to PolyB-fertilized (either inoculated and uninoculated plants) and unfertilized treatments. However, at 75 DAS, the P availability in rhizosphere soil under co-application of PolyB-PSB_Cs_ was enhanced by 15, 92, and 170% compared to OrthoP, PolyP alone and unfertilized plants. As compared to uninoculated treatments, remarkable and significant increase in MBP in the rhizosphere soil of inoculated plants was noted. The MBP was 10-times higher in response to inoculation with PSB_Cs_ compared to all uninoculated plants (regardless the type of P source) at both 30 and 75 DAS.

The increase in P availability in response to dual application of PolyB and PSB_Cs_ can be partly explained by the ability of the PSB_Cs_ to produce significant amounts of organic acids and phosphatases under PolyB application. In that regard, the PolyB-PSB_Cs_ co-application induced a significant increase in rhizosphere soil APase activity at 30 and 75 DAS (Figure 5). The APase activity in the rhizosphere soil of PSB_Cs_-inoculated plants was 1.58-and 3--times higher than PolyB alone and OrthoP (fertilized with readily available P) applications, respectively at the two growth stages (Figure 5).

In addition, the root APase activity was significantly increased in response to PolyB-PSB_Cs_ co-application and P deficiency (P0) (Figure 5). At 30 DAS, the unfertilized plants and PSB_Cs_-inoculated plants showed the highest root APase activity. However, at 75 DAS, root APase activity in inoculated plants was significantly increased by 325 and 120% compared to PolyB alone and OrthoP application. Interestingly, increase of both root and rhizosphere soil APase activities under PolyB-PSB_Cs_ co-application were more pronounced at 75 DAS.

Taken together, dual application of PolyB and PSB inoculation induced remarkable increase of root and rhizosphere soil to promote PolyP hydrolysis and consequently wheat P uptake.

### 3.5. Effect of PolyP-PSB consortium co-application on nutrient contents, yield parameters, and photosynthesis parameters

**Figure 6:**
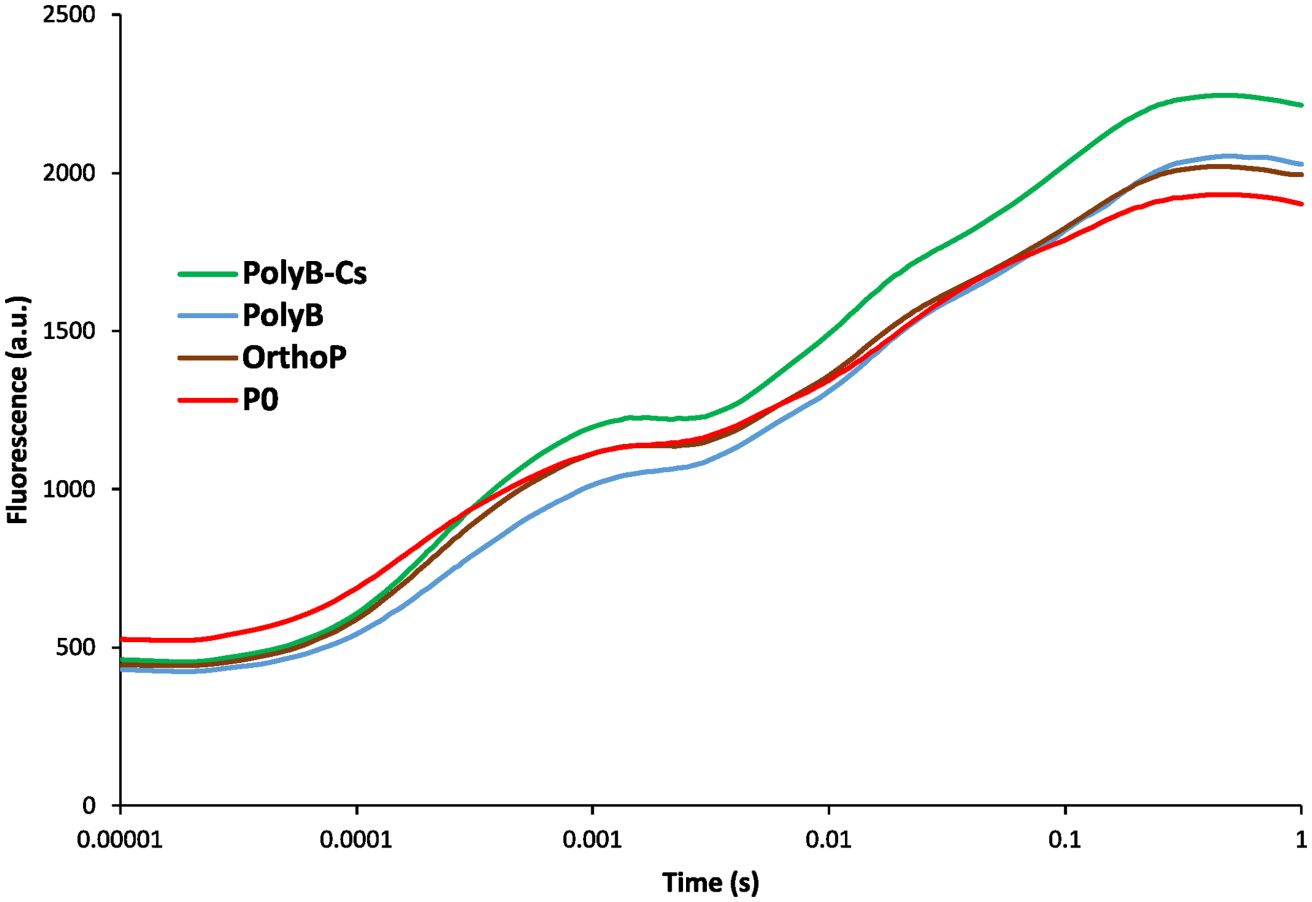
Chlorophyll a fluorescence transient curves of wheat leaves (75 days after sowing) in response to PSB consortium and PolyB co-application. Data are mean values ± SD (*n* = 8). PolyB-Cs, polyphosphate application combined with a consortium of four P-solubilizing bacteria.

In addition to direct effect of PSB and PolyB on wheat P uptake and PUE, the PolyB-PSB_Cs_ co-application positively impacted the wheat aboveground physiological performance including nutrient uptake (N, P, and K), yield related parameters (spike number, spike dry weight, spike N, P, and K contents) and photosynthesis parameters (CCI, PI, Fv/Fm) at 75 DAS (Table 2; Figure 6; Supplementary Figure S1). For instance, the PolyB-PSB_Cs_ co-application significantly enhanced shoot P and K contents by 300 and 65%, respectively, compared to unfertilized plants. Similarly, the PolyB-PSB_Cs_ co-application increased the yield parameters such as spike dry weight (49%), spike number (50%), and spike nutrient contents (N 41%, P 99%, and K 13%) compared to unfertilized plants. Furthermore, the shoot N, P, and K contents to RL ratios were significantly enhanced in response PolyB-PSBCs compared to PolyB alone, indicating that PSB_Cs_ can modulate root traits for better P acquisition (Supplementary Figure S2).

**Table 2:** Effects of PSB consortium and PolyB application on spike dry weight, spike number, nitrogen, phosphorus and potassium contents in shoots and spikes of wheat plants 75 days after sowing.

P and PSB treatments	Shoot N content (mg/plant)	Shoot P content (mg/plant)	Shoot K content (mg/plant)	Spike DW (g)	Spike number (number/pot)	Spike N content (mg/plant)	Spike P content (mg/plant)	Spike K content (mg/plant)
PolyB-Cs	125.22^a^	14.47^ab^	159.28^ab^	2.23^a^	4.43^a^	86.31^a^	10.95^a^	38.38^ab^
PolyB	127.93^a^	14.7^a^	155.58^a^	1.93^ab^	3.75^b^	88.34^a^	10.35^a^	36.12^bc^
OrthoP	133.88^a^	11.77^b^	133.62^b^	1.77^ab^	3.63^b^	72.39^a^	7.8^b^	40.18^a^
P0	93.27^b^	3.62^c^	96.18^c^	1.45^b^	2.95^c^	61.1^a^	5.48^b^	34.01^c^

PolyB-Cs, polyphosphate application combined with a consortium of four P-solubilizing bacteria. Data are means (*n* = 8). Different letters indicate a significant difference according to Tukey’s test: *p* < 0.05.

Inoculation with PSB_Cs_ positively influenced photosynthesis related parameters such as chlorophyll fluorescence, CCI, PI, and Fv/Fm (Figure 6; Supplementary Figure S1). The fertilized plants (regardless of the P source) showed no significant difference in minimal chlorophyll fluorescence (*F*_0_), while the unfertilized plants exhibited the highest *F*_0_. However, at the maximal chlorophyll fluorescence level (F_m_), the transient curves indicated a significant increase of chlorophyll fluorescence in response to PolyB-PSB_Cs_ co-application compared to unfertilized plants, indicating that photosynthesis apparatus is sensitive to P availability and uptake. Similarly, the co-application of PolyB-PSB_Cs_ significantly increased PI (90%), Fv/Fm (108%), and CCI (53%) compared to unfertilized plants.

## 4. Discussion

The present work is among the first studies to assess the contribution of PSB inoculation to improve plant growth performance and PUE of wheat plants under PolyP application. Our findings revealed that PolyB-PSB_Cs_ co-application significantly increased wheat P acquisition, PUE, PFUE, and the whole aboveground plant performance (photosynthesis, biomass production, and yield parameters), with the best results at 75 DAS. The positive effects due to the PolyB-PSB_Cs_ co-application can be partly explained by the capacity of PSB_Cs_ to modulate the trade-offs between morphological and physiological root traits depending on the growth stage and available P status in the rhizosphere. In addition, the enhanced APase activities in root and rhizosphere soil as well as the P availability in rhizosphere soil under PolyB-PSBCs co-application can be a confirmation of the hypothesis that PSB_Cs_ efficiently contribute to PolyP hydrolysis in the soil–plant system (owing to the synergistic effects of PSB species used in this consortium), which leads to better availability and acquisition of P from PolyP.

### 4.1. Dual application of PSB consortium and PolyP enhanced plant growth and plant Pi content

Given the considerable capacity of PSB_Cs_ to increase P availability form PolyP and promote plant growth, our findings confirmed that PolyP-PSB_Cs_ co-application can increase biomass and P uptake of durum wheat plants (Figures 1, 2). These findings were consistent with a previous study demonstrating that PolyP-PSB co-application significantly improved the RDW and SDW of wheat plants at early growth stages (Khourchi et al., 2022a). In addition, the SDW under PolyB-PSB_Cs_ co-application was significantly higher at 75 DAS than at the 30 DAS growth stage, indicating that PSB_Cs_-inoculated plants invest more in shoot biomass production, which may contribute to greater yield (Magney et al., 2016).

The findings of this present study showed that both shoot and root Pi contents were significantly increased at two stages in response to PolyB application, with the highest Pi contents in response to PolyB-PSB_Cs_ co-application at both growth stages (Figure 2). The present findings suggest that PSB_Cs_ can significantly enhance PolyP use efficiency through facilitating P release and plant P acquisition, which is consistent with previous studies indicating that application of PolyP improved shoot and root Pi contents and this improvement was more prominent in response to PSB inoculation (Khourchi et al., 2022a,b).

Given the limited knowledge on PolyP-PSB co-application and the involvement of PSB in PolyP hydrolysis, previous studies demonstrated that co-application of sparingly available P fertilizers and PSB (especially a consortium) significantly increased plant biomass and plant P acquisition (Rahi et al., 2010; Elhaissoufi et al., 2020; Rawat et al., 2022) can be extrapolated to PolyP. For instance, Rahi et al. (2010) found that inoculation with a PSB_Cs_ (*Burkholderia gladioli* 10,216, *Burkholderia gladioli* 10,217, *Enterobacter aerogenes* 10,208, and *Serratia marcescens* 10,238) significantly enhanced shoot Pi contents by 165 and 73%, respectively, compared to uninoculated plants and tricalcium-P-fertilized plants.

### 4.2. Dual application of PSB consortium and PolyP improved wheat PUE and PolyP use efficiency

Regarding the P use efficiency, the results of present study showed that wheat PUE parameters was significantly enhanced, especially at 75 DAS, which indicates that PSB_Cs_ inoculation significantly contribute to increase the roots’ capacity to take up the available P and its allocation to shoots. Plant P acquisition is often linked to the capacity of a crop plant to explore the soil for resources (Shen et al., 2013), and this was clearly noted at 75 DAS, where the shoot Pi to RL ratio was three-times higher in response to PSB_Cs_ compared to unfertilized plants (Figure 3). Similarly, the PFUE was significantly higher under PolyB compared to control (Figure 4), indicating that PolyB could be an efficient source of P for crop nutrition. These results proposed that increased PUE under PolyB-PSB_Cs_ co-application can be attributed to the ability of PSB species to hydrolyze PolyP in the root vicinities and modulate the root traits that are likely involved in P acquisition. In line with that, Khourchi et al. (2022a) reported that PolyP-PSB co-application, notably PSB_Cs_, promoted root hair proliferation, increased root hair length, and influenced root morpho-physiological traits (RL, RSA, and root APase activity) at seedling stage.

### 4.3. Inoculation with PSB consortium significantly enhanced PolyP use efficiency through influencing belowground traits

Our findings showed that enhanced wheat P uptake can be linked to the capacity of both PSB and PolyP to influence the tradeoffs between morphological and physiological root traits (Table 1), which clearly indicates the capacity of PolyP and PSB to modulate root growth depending on the growth stages (Khourchi et al., 2022a; Loudari et al., 2022a). The observed variation in the effects of PSB across the different growth stages can be due to changes in the quality and quantity of root exudates serving as energy sources for these beneficial microbes (Sasse et al., 2018; Chai and Schachtman, 2022). In agreement with our results, inoculation with both individual PSB species and the consortium significantly increased morphological root traits (RL and RSA) and root hair length of wheat fertilized with PolyP (Khourchi et al., 2022a). Moreover, vigorous root morphology in the unfertilized plants at 75 DAS can be explained as a typical response to P deficiency allowing maximal soil exploitation (Wen et al., 2019).

In addition to modulating effects of PSB and PolyP on root growth, the capacity of rhizosphere microbes, including PSB, to hydrolyze PolyP in soil–plant system is still almost neglected when studying PolyP (Khourchi et al., 2023). In that regard, our findings showed that PolyB-PSB_Cs_ co-application significantly enhanced P availability from PolyB through inducing high APase activities in both roots and rhizosphere soil compared to uninoculated and PolyB-fertilized plants (Figure 5). The enhanced MBP under PolyB-PSB co-application confirms the activity of the used PSB_Cs_ and its role in P cycling and turnover from different P pools. The level of immobilized P as MBP varied depending on the turnover rate. In that regard, several previous studies found that the level of immobilized P from MBP varied depending on the turnover rate, which is relatively fast in most cases (a few days to a couple of months depending on soil type and climatic factors) (Achat et al., 2010; Zhang et al., 2014; Zheng et al., 2017; Chen et al., 2021; Elhaissoufi et al., 2022). These studies proposed that MBP can be an important P pool that is potentially available for plant uptake in the short-term.

The increased rhizosphere P availability could be attributed to the synergistic PolyP-hydrolyzing capacities of the four PSB species forming the PSB_Cs_ and their abilities to produce phosphatases and organic acids (Khourchi et al., 2022a). Our findings confirmed that used PSB_Cs_ induced significant increase in APase activity in the rhizosphere soil and roots (Figure 5). In line with our findings, it has been reported that PSB_Cs_ significantly hydrolyze PolyP (with various chain lengths) through exuding significant amounts of P-hydrolyzing enzymes and organic acids *in vitro*, indicating the importance of P-hydrolyzing enzymes, and potentially other metabolites such as organic acids, in P availability from PolyP (Khourchi et al., 2022a). At the soil level, the same study reported that soil APase activity was 2-fold higher in response to co-application of PolyP-PSB compared to the unfertilized plants.

Altogether, our findings strongly suggest that wheat plants under PolyB-PSB_Cs_ co-application adopted trade-offs and balances between root morphological and physiological traits depending on the P availability and plant growth stage (Wen et al., 2019; Khourchi et al., 2022b, 2023). These beneficial effects on root growth and P availability can result in increased plant growth performance through coordination between root and shoot growth.

### 4.4. Dual application of PSB consortium and polyphosphates enhanced nutrient uptake and photosynthesis

The co-application of PolyB-PSB_Cs_ promotes wheat growth performance not only through improving the belowground traits and P acquisition, but also by positively impacting nutrient uptake, yield related parameters, and photosynthesis performance (Table 2; Figure 6; Supplementary Figure S1). The improved total P and K uptake in response to PolyB-PSB_Cs_ suggests that the beneficial effects of PSB_Cs_ on belowground traits can result in enhanced nutrient uptake and crop yield. The inoculated plants exhibited a significant increase in yield parameters (e.g., spike number, spike dry weight, spike P, and K contents). To our knowledge, there are no studies investigating the effect of PolyP-PSB co-application on nutrient uptake and crop yield. However, it is well documented that co-application of various PSB and sparingly available P forms (tricalcium P, dicalcium P, rock P, etc.) significantly increased nutrient uptake and yield of different crops (reviewed in Bargaz et al., 2021). Moreover, the PolyB-PSB_Cs_ co-application increased N, P and K: RL ratios (Supplementary Figure S2), suggesting that PolyB-PSB_Cs_ co-application can improve nutrient acquisition through influencing coordination between above-and below-ground traits.

Considering the crucial role of P in photosynthesis(Wu et al., 2006; Veneklaas et al., 2012), the enhanced P acquisition in response to PolyB-PSB_Cs_ co-application can partly explain the improved photosynthesis parameters (chlorophyll fluorescence, CCI, PI, and Fv/Fm). Although there are no existing studies evaluating the PolyP-PSB co-application on photosynthesis performance, recent studies have reported that PolyP application positively impacts photosynthesis performance by improving P acquisition (Chtouki et al., 2021, 2022; El-Mejjaouy et al., 2022; Loudari et al., 2022b). Similarly, several studies have found that PSB combined or not with P fertilizers can improve photosynthesis as an indirect plant growth promoting effect by optimizing P acquisition efficiency (Dawwam et al., 2013; Zhou et al., 2017; Elhaissoufi et al., 2020; Seyahjani et al., 2020; De Zutter et al., 2022), which can be extrapolated to the findings of the present study.

## 5. Conclusion

The present study reveals that inoculation with PolyP-hydrolyzing PSB_Cs_ enhances PolyP use efficiency, wheat P acquisition, and PUE through modulating belowground (root and rhizosphere) traits involved in both PolyP hydrolysis and P acquisition. More specifically, the co-application of PolyB-PSB_Cs_ significantly induces high acid phosphatase activities at 75 DAS, notably in roots, which were likely exuded into the rhizosphere and contributed to enhancing soil P availability and acquisition from PolyP. These results strongly support the involvement of P-hydrolyzing enzymes in PolyP hydrolysis at the soil–plant level. In addition, PolyP-PSB_Cs_ co-application significantly influences root growth depending on the plant growth stage, and this is clearly indicated by the trade-offs between the morphological and physiological root traits from 30 to 75 DAS to optimize soil P exploration and meet the plants’ P requirements. Moreover, the beneficial effects of PSB_Cs_ on PUE and root growth resulted in the improvement of other aboveground morpho-physiological traits (e.g., plant biomass, nutrient allocation to shoots and spikes, and photosynthesis performance), suggesting that PSB_Cs_ co-applied with PolyB likely stimulates stronger above/belowground trait coordination allowing better growth performance and crop productivity.

The above findings confirm the importance of combining a PSB inoculant (with a high ability to produce P-hydrolyzing enzymes, especially under soil conditions) with PolyP as an integrated and innovative P application approach to enhance PUE and crop productivity. However, more studies focusing on the composition of root exudates and rhizosphere available P status in response to the co-application of PolyB and PSB_Cs_ should be considered in future research. Additional inoculation experiments under both controlled and field conditions are needed to confirm the observed effects under controlled conditions and elucidate the contribution of both rhizosphere processes (e.g., phosphatase activities, organic acid exudation, and rhizosphere acidification) and PolyP-hydrolyzing microbes in enhancing PolyP use efficiency. Moreover, further studies are also needed to uncover the potential beneficial effects of PolyP and PSB/arbuscular mycorrhizal fungi co-application on plant nutrient acquisition, crop productivity, and rhizosphere microbiota composition under various pedo-climatic conditions.

## Data availability statement

The original contributions presented in the study are included in the article/Supplementary material, further inquiries can be directed to the corresponding authors.

## Author contributions

SK and AB conceived and designed the experiments and wrote the first version of the manuscript. SK conducted the experiments. SK, WE, AI, MH, and RG provided technical assistance in plant and soil analyses. SK, AB, PD, and YZ discussed the data, contributed to the writing and reviewing of manuscript drafts. All authors reviewed and approved the submitted version.

## Funding

The SoilPhorLife Program sponsors (grant ID SoilPhorLife N°5); namely OCP Group and Prayon, are greatly acknowledged for funding this study.

## Conflict of interest

The authors declare that the research was conducted in the absence of any commercial or financial relationships that could be construed as a potential conflict of interest.

## Publisher’s note

All claims expressed in this article are solely those of the authors and do not necessarily represent those of their affiliated organizations, or those of the publisher, the editors and the reviewers. Any product that may be evaluated in this article, or claim that may be made by its manufacturer, is not guaranteed or endorsed by the publisher.
