# Supplementary material for: Integrated use of polyphosphate and P-solubilizing bacteria enhanced P use efficiency and growth performance of durum wheat
Source: Front Microbiol. 2023 Jul 5;14:1211397. doi: 10.3389/fmicb.2023.1211397 (PMC10354339; doi:10.3389/fmicb.2023.1211397)
Supplement: Supplementary file 2 [file Data_Sheet_1.docx]

**Supplementary material**


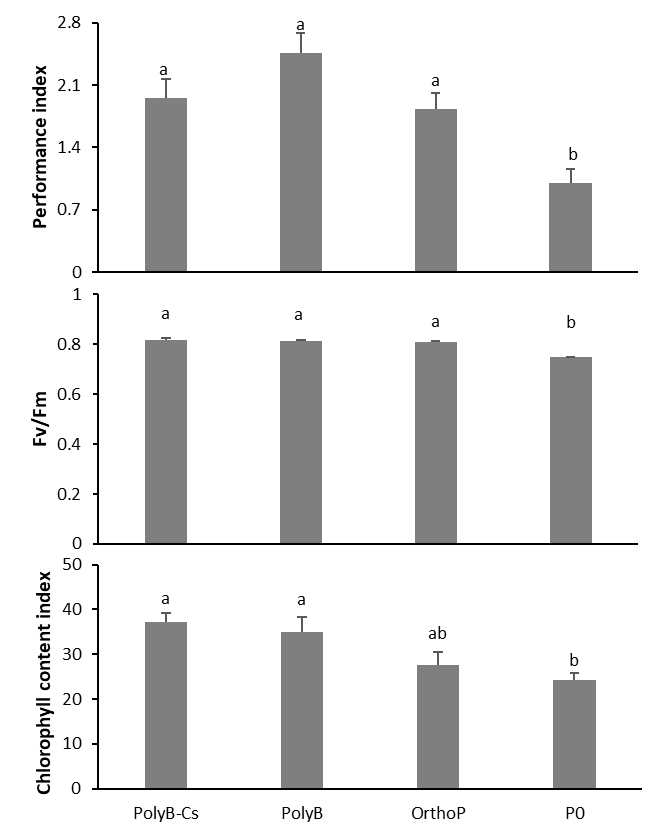


**Figure S1**: Effects of PSB consortium and PolyB application on chlorophyll content index and chlorophyll fluorescence parameters (Fv/Fm: potential quantum efficiency of PSII and PI: performance index) of wheat leaves at 75 days after sowing. Data are mean values ± SD (n=8), Different lowercase letters above the bars indicate significant differences (p < 0.05) according to Tukey’s test. PolyB-Cs: polyphosphate application combined with a consortium of four P-solubilizing bacteria.


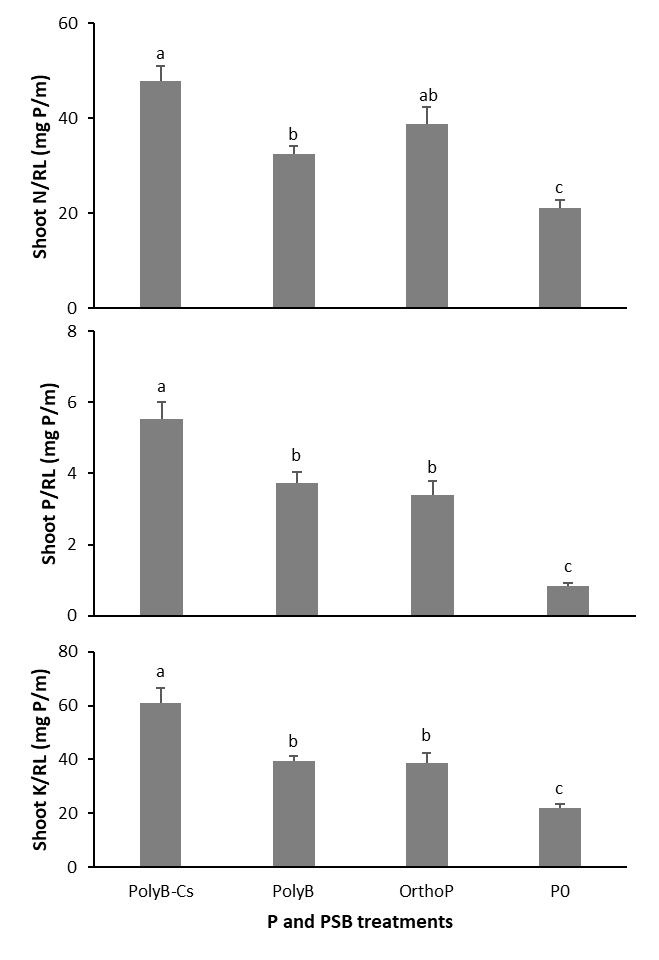


**Figure S2**: Effects of PSB consortium and PolyB application on shoot P/RL, shoot N/RL and shoot K/RL of wheat plants at 75 days after sowing. Data are mean values ± SD (n=6), Different lowercase letters above the bars indicate significant differences (p < 0.05) according to Tukey’s test. PolyB-Cs: polyphosphate application combined with a consortium of four P-solubilizing bacteria.
